# Tumor‐associated DNA mutation detection in individuals undergoing colonoscopy

**DOI:** 10.1002/cam4.1249

**Published:** 2017-11-10

**Authors:** Phillip Fleshner, Glenn D. Braunstein, Gayane Ovsepyan, Theresa R. Tonozzi, Anja Kammesheidt

**Affiliations:** ^1^ Division of Colorectal Surgery Cedars‐Sinai Medical Center Los Angeles California 90048; ^2^ Pathway Genomics San Diego California 92121

**Keywords:** Circulating free DNA, circulating tumor DNA, colonoscopy, colorectal cancer, colorectal polyps, liquid biopsy, mutation

## Abstract

The majority of colorectal cancers (CRC) harbor somatic mutations and epigenetic modifications in the tumor tissue, and some of these mutations can be detected in plasma as circulating tumor DNA (ctDNA). Precancerous colorectal lesions also contain many of these same mutations. This study examined plasma for ctDNA from patients undergoing a screening or diagnostic colonoscopy to determine the sensitivity and specificity of the ctDNA panel for detecting CRC and precancerous lesions. Two hundred patients without a history of nonskin cancer had blood drawn before a colonoscopy. Plasma ctDNA was measured with a 96 mutation panel for nine cancer driver genes. The ctDNA results were correlated with the findings at colonoscopy. Of the 200 patients, 176 (88%) had wild‐type DNA, 12 (6%) had mutations detected, and 12 (6%) had indeterminate results. Colonoscopy was normal in 80% of the patients and 20% were found to have polyps. No CRC was found in this study, precluding a determination of true‐positive rate for CRC detection. Our ctDNA panel was positive in 13.2% of patients with colonic polyps found at colonoscopy, while 4.7% of patients with normal colonoscopy also had ctDNA detected, which may represent ctDNA released from a benign process, an occult tumor, or an acquired somatic mutation from clonal hematopoiesis.

## Introduction

Colorectal carcinoma (CRC) is the third most common nonskin cancer in men and women in the United States, and the third leading cause of cancer deaths in women and the second in men 1. In a male, the lifetime risk is 1 in 21 (4.7%), in a female it is 1 in 23 (4.4%), and the prevalence of CRC in an unscreened population with average risk is 0.5–1% 1. The death rate has decreased steadily over the last 3 decades in part due to screening for CRC, removal of premalignant polyps, and improved treatment 1, 2.

Several modalities are available to screen for CRC and dysplastic polyps. These include guaiac‐based fecal occult blood tests, fecal immunochemical test (FIT), flexible sigmoidoscopy, double‐contrast barium enema radiography, CT colonography (virtual colonoscopy), and colonoscopy. Each of these has advantages and disadvantages, with colonoscopy being shown to decrease both CRC incidence and mortality, but having diminished patient acceptance in comparison to FIT 3, 4, 5. However, sensitive stool guaiac tests and immunochemical tests have reduced sensitivity and specificity for detecting CRC and advanced adenomas 6.

In addition to hemoglobin, other exfoliated markers of colonic neoplasia have been examined in feces 6. One of the best studied is a multitarget DNA test that examines a stool specimen for aberrantly methylated *BMP3* and *NDRG4* promoter regions, mutant *KRAS*, and *β*‐actin, along with an immunochemical test for hemoglobin 7. In a study involving almost 10,000 patients, 0.7% had colorectal cancer and 7.6% had advanced precancerous lesions on colonoscopy. The multigene test detected 92.3% of the patients with CRC and 42.4% of patients with advanced precancerous lesions, while FIT detected 73.8% of patients with CRC and 23.8% with advanced precancerous lesions 7. However, 10.2% of patients with the DNA testing had false‐positive results, while 3.6% false‐positive findings occurred with FIT 7.

Small quantities of circulating free DNA (cfDNA) can be detected in the plasma from healthy individuals 8. Elevated levels are found in the blood of patients with inflammatory diseases as well as with cancer, including CRC 8, 9, 10, 11, 12, 13, 14. The majority of cancers, including CRC, harbor somatic mutations and epigenetic modifications which are associated with the activation, progression, and metastasis of the tumors 15, 16. Several mutations have been identified in CRC and precancerous colorectal lesions in both the tumor tissue and circulation as circulating tumor DNA (ctDNA). These include mutations in *KRAS*,* TP53*,* APC*,* BRAF*, and epigenetic alterations in *HLTF*,* HPP1*,* hMLH1*,* TAC1*,* SEPT9*,* NELL1*,* AGBL4*,* FLI1*,* TWIST1*,* SST*,* p16INK4a*, and *RASSF1A* 11, 14, 17, 18, 19, 20, 21, 22, 23, 24, 25, 26, 27, 28, 29, 30, 31, 32, 33, 34, 35, 36, 37.

Recently, we detected mutations in *CTNNB1*,* EGFR*,* GNAS*,* KRAS*,* TP53*, and *PIK3CA* in the plasma from patients with CRC, using a 96 ctDNA mutation panel for nine cancer driver genes 38. In the patients tested with this assay, the detection rate was 24.0% (14.7% stage I, 18.8% stage II, 33.3% stage III, 50.0% stage IV), with 50% of colon cancer patients detected (stages I–IV) 38. In addition, data from COSMIC 39, irrespective of cancer stages, suggest that our theoretical detection rate with the 96 panel should be around 66%. Of 778,342 variants detected in 46,124 colon samples, our assay would have been able to detect 33,828 variants in 30,565 samples if the tumor shed minimally detectable amounts of ctDNA into the circulation. In order to determine the usefulness of this panel for screening patients for CRC or colorectal precancerous lesions, we initiated a trial in which plasma samples were obtained in 200 patients without known cancer before they underwent a colonoscopy and correlated the ctDNA results with findings at colonoscopy.

## Methods

### Patients and protocol

A study cohort of 200 patients, comprising 120 (60%) males and 80 (40%) females between the ages of 26 and 84 years with a mean age of 60 years scheduled to undergo a screening or diagnostic colonoscopy, were recruited to the study (Table 1). Eighty‐five percent were Caucasian, 4% Hispanic, 2.5% African American, 1% Asian, and 7.5% not reported. The only exclusion criterion was a history of prior cancer, except for basal cell carcinomas of the skin. Consenting patients completed a questionnaire regarding risk factors for colorectal neoplasms. None had a known germline mutation associated with colorectal carcinoma. Each had a ~20 mL blood sample drawn into cfDNA BCT blood collection tubes (Streck, Omaha, NE, USA) before the colonoscopy. Blood collection tubes were typically received at the laboratory within 1–3 days of the blood draw. The protocol was approved by Chesapeake IRB (Columbia, MD, USA) and was registered at ClinicalTrials.gov (ClinicalTrials.gov Identifier: NCT02665299).

**Table 1:** Patient characteristics and indications for colonoscopy

	Total group (*n* = 200)	With or without mutations (*n* = 188)	Indeterminate (*n* = 12)
Age (years; range)	60.2 (26–84)	60 (26–84)	63 (45–80)
Gender			
Male	120 (60%)	112 (59.6%)	8 (66.7%)
Female	80 (40%)	76 (40.4)	4 (33.3%)
Ethnicity			
African American	5 (2.5%)	5 (2.7%)	0
Asian	2 (1%)	2 (1%)	0
Caucasian	170 (85%)	158 (84%)	12 (100%)
Hispanic	8 (4%)	8 (4.3%)	0
Not reported	15 (7.5%)	15 (7.8%)	0
Indication for colonoscopy			
Abdominal pain	44 (22%)	41 (21.8%)	3 (25%)
Family history	12 (6%)	12 (6.4%)	0
History of polyps	56 (28%)	52 (27.7%)	4 (33.3%)
Rectal bleeding	30 (15%)	28 (14.9%)	2 (16.7%)
Screening	44 (22%)	41 (21.8%)	3 (25%)
Other^a^	14 (7%)	14 (7.4%)	0

a Other includes: anal abscess (1); Crohn's disease (7); constipation (1); diarrhea (2); diverticulitis (1); inflammatory bowel disease (1); ulcerative colitis (1).

### Circulating tumor DNA measurement

To separate plasma, both blood tubes were spun for 10 min at 2000*g* at room temperature, plasma layers were combined into a new tube, respun for 10 min at 2000*g* at 4°C, then transferred again and frozen in 5 mL plus residual volume aliquots. As previously described, cfDNA was isolated from 5 mL of plasma using the QIAmp Circulating Nucleic Acid Kit (Qiagen, Hilden, Germany), and the cfDNA yield determined with the Qubit dsDNA HS Assay Kit (Thermo Fisher Scientific, Waltham, MA, USA) 38. Library preparation was performed with 10–300 ng input DNA. The ctDNA was analyzed with the 96 mutation assay (CancerIntercept, Pathway Genomics, San Diego, CA, USA) which uses polymerase chain reaction amplification and mutation enrichment based on a multiplexed detection technology 40. During the enrichment process wild‐type DNA is removed which lowers the necessary sequence depth. Next‐generation sequencing was performed using Illumina MiSeq (Illumina, San Diego, CA, USA). CancerIntercept detects 96 mutations in nine cancer driver genes (*BRAF*,* CTNNB1*,* EGFR*,* FOXL2*,* GNAS*,* KRAS*,* NRAS*,* PIK3CA*, and *TP53*). The assay has a >78% analytical sensitivity for 2–5 copies, >98% for 5–9 copies, and 100% for >9 copies across the cfDNA input range of 10–300 ng 38.

### Data analysis

Using the results of the colonoscopy as the clinical benchmark, the comparative utility of the time‐matched cfDNA and ctDNA results was analyzed. Student's *t*‐test was used to compare groups with a *P* ≤ 0.05 being considered significant.

## Results

The colonoscopies were performed for a variety of indications ranging from screening to colonoscopies for abdominal symptoms (Table 1).

Mutations were detected in the circulation of 12 (6%) patients, while wild‐type DNA was noted in 176 (88%) patients, and in 12 (6%) patients the results were indeterminate (Table 2). Results were classified as “indeterminate” if the initial result was between 2 and 5 copies and could not be independently verified with a second blood sample prior to colonoscopy. Patient characteristics and indications for colonoscopy were similar among these 12 patients with indeterminate results as with the other 188 subjects (Table 1). Patients with indeterminate results were excluded from further analysis.

**Table 2:** Colonoscopy result and liquid biopsy result

	Total (*n* = 188)^a^			
	Negative (wild type)		Positive (mutation detected)	
	Participant number (*n* = 176)		Participant number (*n* = 12)	
Result		%		%
Liquid biopsy result				
Wild type	176	93.6		
Mean cfDNA (SEM), ng/mL	57.6 (1.6)			
Mutations detected			12	6.4
Mean cfDNA (SEM), ng/mL			73.8 (7.4)	
Number of mutations detected			14	
Colonoscopy result				
Normal (total *n*=150)	143	81.3	7	58.3
Mean cfDNA (SEM), ng/mL	57.4 (1.8)		80.7 (9.2)	
Polyp (total *n*=38)	33	18.8	5	41.7
Mean cfDNA (SEM), ng/mL	58.1 (4.1)		64.2 (12.0)	
Polyp size	*n* = 33		*n* = 5	
<1cm	33	100	4	80.0
1–2cm	–		1	20.0
>2cm	–		–	
Polyp type	*n* = 33		*n* = 5	
Hyperplastic	12	36.4	–	
Inflammatory	2	6.1	1	20.0
Leiomyoma	1	3.0	–	
Sessile	2	6.1	–	
Adenoma				
Tubular	15	45.5	2	40.0
Tubulovillous	1	3.0	2	40.0

SEM, standard error of the mean.

a Table excludes 12 indeterminate samples.

Colonoscopy was normal in 150 (80%) of the 188 patients and polyps were noted in 38 (20%) patients (Table 2). No patient was found to have CRC despite many of the patients being at increased risk because of a strong family history of CRC or a personal history of colonic polyps. ctDNA was found in seven patients with normal colonoscopies (95.3% specificity with a 4.7% false‐positive rate) and five patients with polyps (13.2% true‐positive rate) resulting in an 86.8% false negative rate. The average age of the ctDNA‐positive patients was 67 years (range 52–83), and 44% were male and 58% female. Raising the detection limit from two or more copies of mutant DNA to five or more copies decreased the sensitivity to 10.5% and slightly raised the specificity to 96%. At a level of 10 or more copies, the sensitivity was 2.6% and specificity 96.7%. Most of the polyps found at colonoscopy were <1 cm in both patients with or without detectable ctDNA. The majority of the polyps were tubular adenomas followed by hyperplastic polyps.

The mean cfDNA level for 5 mL of plasma was similar in 150 patients without polyps and the 38 patients with polyps regardless of their mutation status (58.53 ng/mL vs. 58.9 ng/mL, N.S.). However, the mean cfDNA level was slightly higher in patients with ctDNA detected than in those without ctDNA (73.8 ng/mL vs. 57.6 ng/mL, *P* = 0.0124). cfDNA levels were higher in patients with polyps and ctDNA than in those with polyps without ctDNA being detected (64.2 ng/mL vs. 58.1 ng/mL), but the results were not significantly different (*P* = 0.6).

Table 3 lists the specific mutations found in the 12 patients in whom ctDNA was detected.

**Table 3:** Circulating tumor DNA (ctDNA) levels and percent abundance mutant DNA relative to circulating free DNA (cfDNA) in participants with a mutation detected

Subject	Mutation	Copy number	Abundance mutant ctDNA relative to input cfDNA (%)	Pathology result
1	BRAF_e15_K601E	5.5	0.052	TA
2	BRAF_e15_V600E	2.4	0.019	N
	GNAS_e8a_R201H	12.3	0.097	
3	GNAS_e8a_R201C	5.5	0.022	I
4	GNAS_e8a_R201C	4.7	0.018	TA
5	GNAS_e8a_R201H	14.6	0.081	N
6	GNAS_e8a_R201H	29.7	0.110	N
7	GNAS_e8a_R201H	5.2	0.049	TVA
8	GNAS_e8a_R201H	4.5	0.020	N
9	KRAS_e2a_G13D	12.2	0.050	TVA
10	NRAS_e2a_G12D	9.6	0.032	N
11	NRAS_e2a_G12D	85.9	0.326	N
	TP53_e8a_R273H	13.7	0.052	
12	TP53_e8a_R273H	12.7	0.037	N
Mean		15.6	0.069	

I, inflammatory; N, normal; TA, tubular adenoma; TVA, tubulovillous adenoma.

## Discussion

Despite the estimated prevalence of CRC in patients with average risk undergoing colonoscopy being 0.5–1%, none of the 200 patients in our study were found to have CRC. Thus, we are unable to determine the true‐positive detection rate for CRC of our ctDNA panel. Kopreski and coworkers prospectively collected plasma from 240 patients undergoing colonoscopy, and found eight patients with CRC. Five of these patients had *KRAS* mutations in their tumor tissue and all five had the same mutations detected in their plasma, giving a 62.5% true‐positive rate 24. In another screening study, Perrone and colleagues found 12 CRC, and 1 (8%) had a *KRAS* mutation found in their blood 14. Several investigators measured plasma methylated *SEPT9* DNA in patients who were about to undergo colonoscopy. Warren et al. did not find CRC in the 300 colonoscopy patients from a community clinic, and therefore, could not provide sensitivity data for CRC detection 29. In contrast, Church and coauthors found 53 CRC in 6874 patients entered into the PRESEPT study, and 27 (50.9%) had methylated *SEPT9* detected in their plasma 30.

In addition to CRC, there are colonic neoplasms that are premalignant, many of which exhibit oncogene mutations, microsatellite instability, and methylation epigenetic changes 19, 41, 42, 43. These lesions include hyperplastic polyps, tubular adenomas, tubulovillous adenomas, villous adenomas, traditional serrated adenomas, sessile serrated adenomas/sessile serrated polyps, and hamartomatous polyps 19. In our series, we found that 38/188 (20%) patients had one or more of these lesions. Of that group, 5/38 (13.2%) had detectable levels of ctDNA. Similar findings have been noted by other investigators examining the utility of ctDNA measurements in screening for CRC or premalignant colon neoplasms. Kopreski and colleagues found 62 (25.8%) polyps and 65 (27%) non‐neoplastic tissue including hyperplasia, colitis, or nondiagnostic histopathology in their 240 patients 24. Twenty‐two (33.8%) of the 62 patients with adenomas and 9/65 (13.8%) of those with hyperplastic or other non‐neoplastic lesions had *KRAS* mutations in their plasma. In their prospective colonoscopy study, Perrone and coworkers found 22 instances of high‐grade intraepithelial neoplasia in adenomas (12.9%), 54 adenomas (31.8%), and 19 hyperplastic lesions (11.2%) in the 170 patients. *KRAS* mutations were found in the plasma of 3/19 patients with high‐grade intraepithelial neoplasia (15.8%), 1/54 patients with adenomas (1.8%), and none of the patients with hyperplasia 14. In the study of Warren et al., 104 (34.7%) of the 300 patients had adenomas, and 38 (12.7%) had hyperplastic or other polyps. Of these, circulating methylated *SEPT9* was found in 12 (11.5%) patients with adenomas and 1 (2.6%) of the patients with other polyps 29. In the PRESEPT study, Church and coworkers found 666 (9.7%) advanced and 2359 (34.3%) nonadvanced adenomas in the 6874 patients who underwent colonoscopy 30. They found circulating methylated *SEPT9* in 9.6% of the advanced adenomas and 7.7% of the nonadvanced adenomas. Since these polyps are potentially premalignant and should be excised, their detection through measurement of ctDNA should be useful and the finding of a positive test might increase the rate of screening colonoscopies, which suffers from poor patient compliance 4, 5.

We found that 7 of 150 patients without lesions found at colonoscopy had detectable ctDNA. This false‐positive rate of 4.7% is similar to that found by others carrying out screening colonoscopy studies. Kopreski et al. noted that 37 (21.8%) of the 170 patients with hyperplasia, non‐neoplastic lesions, or no lesions had circulating *KRAS* mutations in their plasma, for a false‐positive rate of 15.4% (37/240) 24. Warren and colleagues noted that 8/164 (5.7%) of patients with diverticulosis, hemorrhoids, Crohn's disease, or normal colonoscopies had circulating methylated *SEPT9* for an overall false‐positive rate of 4.0% 29. Church and coworkers reported that 8.6% of patients with no evidence of disease had measurable circulating methylated *SEPT9* which extrapolates to a 4.7% false‐positive rate in their study 30. In our prior study of ctDNA detection in healthy individuals, we found a 3.9% false‐positive rate 38.

There are several possible explanations for the false positives noted in our study and that of others. It has been well established that benign diseases, especially inflammatory conditions, may be associated with elevated levels of cfDNA 8, 44. Additionally, somatic DNA mutations that are associated with cancer have been identified in histologically normal skin and colonic mucosa 45, 46, 47. *KRAS* and *APC* mutations also have been identified in aberrant crypt foci in the colon which may be precursors of adenomas and CRC, but require magnifying endoscopy and methylene blue staining for detection 18, 20, 21, 48. Further underscoring the fact that apparently normal colonic mucosa may harbor cancer driver gene mutations, *KRAS* mutations have been found in colonic effluent samples of patients at increased risk of CRC, but with normal colonoscopies 49. The source of the ctDNA could be from a neoplasm outside of the colorectal area. Since patients with a known malignant neoplasm were excluded and patients were contacted 6–12 months after their initial test to determine if their health status had changed, such tumors would have to be occult. We also have speculated that the apparent false positives that we and others have noted in ctDNA studies may represent the detection of DNA released from apoptotic cells or destruction of precancerous cells, benign inflammatory lesions such as endometriosis, and small neoplasms with somatic DNA mutations during the normal process of immune surveillance 38, 50. Genovese et al. demonstrated that there is an age‐related increase in clonal hematopoiesis with somatic mutations including cancer driver genes in the white blood cells of 12,380 individuals unselected for cancer or hematologic phenotypes 51. In their study, 10% of persons older than 65 years exhibit this phenomenon, and a few went on to develop hematologic cancers. Lui and coworkers showed that most of the cfDNA in the plasma is predominantly hematopoietic in origin 52. Since the average age of our patients was 60 years, it is likely that the source of the ctDNA in our patients with normal colonoscopies at least partly represents clonal hematopoiesis. This is one emerging reason for false‐positive results in ctDNA analysis, which is difficult to remedy in the absence of matched buffy coat and matched tissue samples.

There are several limitations to our study. First, no CRC were found in the 200 patients, although many fell within a high‐risk group for developing CRC. This precluded us from determining the true‐positive rate for detecting CRC with our ctDNA panel. Second, we did not subject the polyps that were removed to tissue DNA analysis, and thus, do not know whether the polyps in the 13.2% of the patients with polyps and detectable plasma ctDNA were in fact the source of the ctDNA. Similarly, we did not measure the ctDNA levels following polyp removal to determine if the ctDNA became undetectable. Also, the majority of the polyps detected was <1 cm and may have had little or no malignant potential. Finally, our follow‐up on the patients was a year or less, therefore, we do not know if any of the patients with detectable ctDNA were harboring an occult noncolonic neoplasm at the time of plasma sampling that was the source of the ctDNA.

In conclusion, our ctDNA panel and methodology detected ctDNA in the plasma of 13.2% of patients with colonic polyps detected on colonoscopy. The 4.7% false‐positive rate may reflect ctDNA released from an occult tumor, a benign process, or somatic mutations occurring during the process of clonal hematopoiesis. Studies in much larger cohorts with combined tissue analysis and more comprehensive clinical follow‐up would be required to establish the utility of this assay further for a screening application. The lack of a difference in cfDNA results between those patients with polyps and those without in our cohort indicates that measurements of cfDNA are not useful for screening purposes.

## Conflict of Interest

Braunstein, Kammesheidt, and Tonozzi are employees of Pathway Genomics. Fleshner and Ovsepyan do not have conflict of interest.
